# Magnetic bubblecade memory based on chiral domain walls

**DOI:** 10.1038/srep09166

**Published:** 2015-03-16

**Authors:** Kyoung-Woong Moon, Duck-Ho Kim, Sang-Cheol Yoo, Soong-Geun Je, Byong Sun Chun, Wondong Kim, Byoung-Chul Min, Chanyong Hwang, Sug-Bong Choe

**Affiliations:** 1 Center for Nanometrology, Korea Research Institute of Standards and Science, Daejeon 305-340, Republic of Korea; 2 CSO and Department of Physics, Seoul National University, Seoul 151-742, Republic of Korea; 3 Spin Convergence Research Center, Korea Institute of Science and Technology, Seoul 136-791, Republic of Korea

## Abstract

Unidirectional motion of magnetic domain walls is the key concept underlying next-generation domain-wall-mediated memory and logic devices. Such motion has been achieved either by injecting large electric currents into nanowires or by employing domain-wall tension induced by sophisticated structural modulation. Herein, we demonstrate a new scheme without any current injection or structural modulation. This scheme utilizes the recently discovered chiral domain walls, which exhibit asymmetry in their speed with respect to magnetic fields. Because of this asymmetry, an alternating magnetic field results in the coherent motion of the domain walls in one direction. Such coherent unidirectional motion is achieved even for an array of magnetic bubble domains, enabling the design of a new device prototype—magnetic bubblecade memory—with two-dimensional data-storage capability.

Recent progress in the control of magnetic domain walls (DWs) has suggested a number of prospective opportunities for next-generation DW-mediated devices^1^,^2^,^3^,^4^,^5^. Among these, coherent unidirectional DW motion has been proposed to replace the mechanical motion of magnetic media in hard-disk drives, thereby enabling the creation of a solid-state nonvolatile data-storage device—so-called racetrack memory—with high storage capacity, low power, and high mechanical stability^1^. Such coherent unidirectional motion was first achieved by injecting current into magnetic nanowires^4^,^5^,^6^. In this scheme, current-induced spin-transfer^6^ and spin-orbit torques^7^,^8^ exert forces on DWs by transferring electron spins to the local magnetic moment, resulting in DW motion along the direction of force. It is therefore possible to realize the unidirectional motion of multiple DWs, leading to the recent development of DW shift registers^4^,^5^. Such DW motion, however, requires a high threshold current, which is inevitably accompanied by high Joule heating^9^ that may cause severe artifacts. Structural modulation of the nanowires has therefore been proposed to reduce the threshold current by introducing DW tension^10^. With wedge-shaped modulation, the DW tension exerts a force on the DWs to reduce the tension energy and consequently, facilitates the DW motion toward the apex edge^11^. It has been revealed that periodic structural modulation allows DW-tension-induced unidirectional motion to be solely driven by the magnetic field without any current injection, as demonstrated by magnetic-ratchet shift registers^12^. It has also been demonstrated that vertical composition modulation leads to unidirectional DW motion along the vertical direction^13^. These schemes are, however, extremely sensitive to tiny structural irregularities in the devices and thus, require highly sophisticated nanofabrication processes. The magnetic bubble memory^14^ commercialized in 1970s also utilizes the unidirectional motion of magnetic bubble domains, but it requires tiny magnetic guide patterns onto the films to attract and repel the bubble domains under rotating magnetic field. Here, we demonstrate a new scheme for unidirectional DW motion based on skyrmion-like magnetic bubble domains^15^. This scheme is applicable even to unpatterned films in the absence of any current injection or structural modulation.

The magnetic skyrmion^15^,^16^,^17^,^18^,^19^,^20^ is a topological object in which the internal spins whirl around the core in all directions and thus, shield the core spins from outer spins of the opposite orientation. Magnetic skyrmions have been observed in several helical magnets, where the helical spin alignment is caused by the Dzyaloshinskii-Moriya interaction (DMI)^21^,^22^. Recently, it has been observed that metallic ferromagnetic multilayer films also exhibit finite DMI because of their asymmetric layer structure, resulting in skyrmion-like magnetic bubble domains with a Néel DW configuration^23^,^24^.

## Results

### Single bubble motion

**Figure 1:**
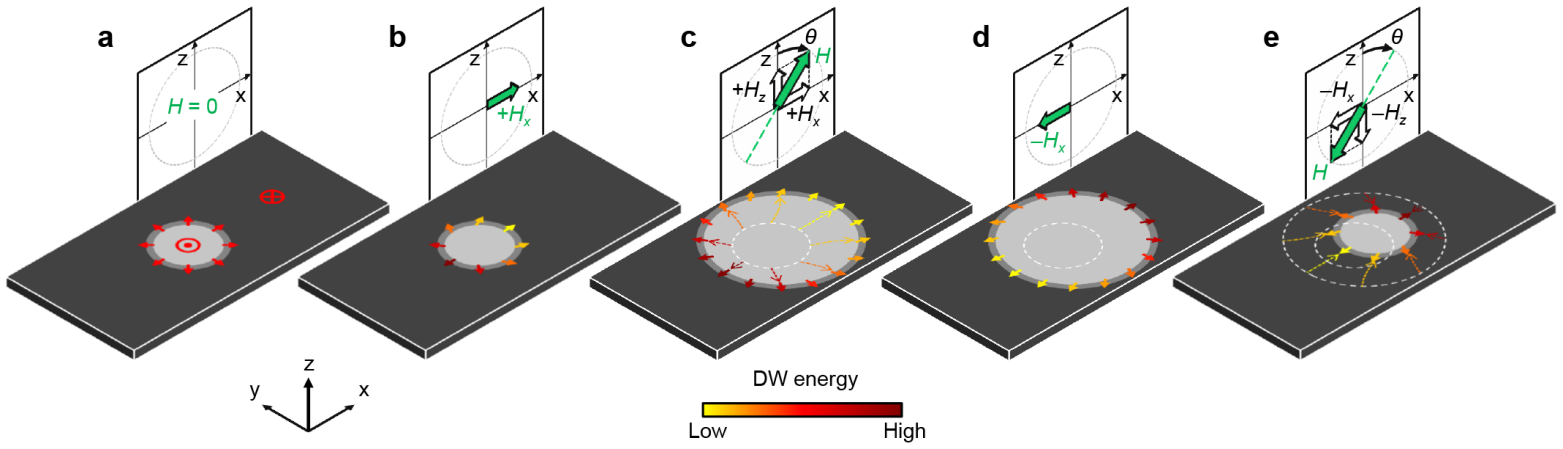
Schematic descriptions of the unidirectional bubble motion. (a) Illustration of a bubble domain (bright circle) and the DW (grey ring), surrounded by a domain of opposite magnetization (dark area). The red symbols and arrows indicate the direction of the magnetization inside the DW and domains. (b) Asymmetric DW-energy distribution under an in-plane magnetic field +*H_x_* (green arrow), as visualized by the color contrast of the arrows on the DW according to the scale bar at the bottom. (c) Asymmetric DW expansion under a magnetic field *H* ( = (+*H_x_*,+*H_z_*)) with a tilting angle *θ*. (d) Asymmetric DW-energy distribution under the reversed in-plane magnetic field −*H_x_*. (e) Asymmetric DW shrinkage under the reversed magnetic field (−*H_x_*,−*H_z_*). The dashed circles represent the previous DW positions.

We demonstrate that a sequence of applying magnetic fields leads to a unidirectional motion of magnetic bubble domains. Figure 1a illustrates a skyrmion-like bubble domain with a Néel DW configuration, where the magnetization 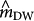 (red arrows) inside the DW is oriented radially outward in all directions. Because of the rotational symmetry with respect to the center of the bubble, the present bubble expands or shrinks circularly under an out-of-plane magnetic field *H_z_* (Ref. 10). However, if one applies an in-plane magnetic field *H_x_*, 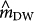 becomes tilted to the direction of *H_x_* due to the Zeeman interaction as illustrated by the arrows in Fig. 1b and thus, the rotational symmetry is broken since 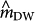 is not oriented radially outward in all directions. The different angle between 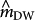 and *H_x_* then results in different Zeeman energy contribution to the DW energy: the parallel alignment has a lower DW energy, whereas the antiparallel alignment has a higher DW energy. Such asymmetric DW energy distribution is shown by the color contrast of the arrow in Fig. 1b. This bubble domain then exhibits asymmetric expansion under *H_z_* (Fig. 1c) because the DW speed depends on the DW energy^25^,^26^,^27^. At this instant, if one reverses the polarity of the in-plane magnetic field (i.e., applies −*H_x_*), then the asymmetry in the DW-energy distribution is also reversed (Fig. 1d). With applying −*H_z_*, this bubble domain shrinks toward a different location from the original position of the domain (Fig. 1e). Consequently, the center of the bubble shifts along the *x* axis from the original position. Such a shift of the center can be continuously generated along the same direction by repeating the process illustrated in Figs. 1c and e, in which collinear magnetic fields (+*H_x_*,+*H_z_*) and (−*H_x_*,−*H_z_*) are alternately applied. Therefore, unidirectional bubble motion can be achieved by applying an alternating magnetic field generated by a single coil that is tilted by an angle *θ* ( = atan(*H_x_*/*H_z_*)) to the film normal.

**Figure 2:**
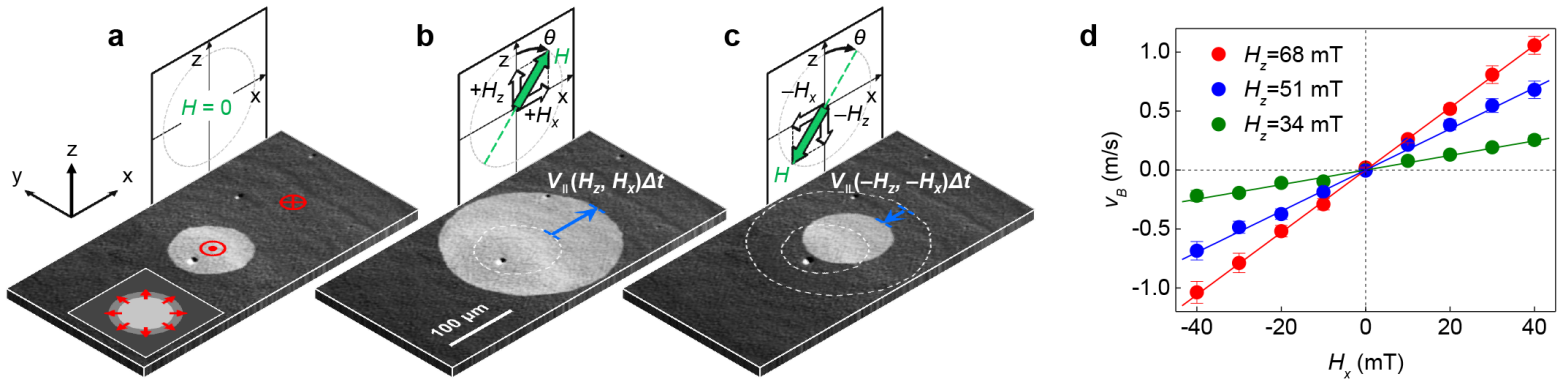
Experimental verification of the unidirectional bubble motion from Pt/Co/Pt film. (a) MOKE image of the initial bubble domain (bright circle) surrounded by a domain of opposite magnetization (dark area). The red symbols indicate the direction of the magnetization in the domains. The inset illustrates the expected 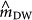 (red arrows) in the right-handed chiral DW configuration. (b) The expanded bubble domain after application of a (+*H_x_*,+*H_z_*) pulse (*H_x_* = 30 mT, *H_z_* = 4 mT, Δ*t* = 100 ms), where Δ*t* is the pulse duration time. The blue arrow indicates the DW displacement *V*_∥_(*H_z_*,*H_x_*)Δ*t*. The dashed circle represents the initial DW position. (c) The shrunken bubble domain after application of a (−*H_x_*,−*H_z_*) pulse. The blue arrow indicates the DW displacement *V*_∥_(−*H_z_*,−*H_x_*)Δ*t*. The dashed circles represent the previous DW positions. (d) Measured *V_B_* with respect to *H_x_* for several *H_z_*. The error bars correspond to the standard deviation from data obtained by sampling 10 times.

The predicted behavior discussed above can be readily verified for Pt/Co/Pt films (see Supplementary Information 1). Recent studies have revealed that these films have a positive DMI and thus exhibit the right-handed chiral DW configuration^25^,^26^. Figure 2a presents an image of a bubble domain captured using a magneto-optical Kerr effect (MOKE) microscope (Ref. 10 and see Supplementary Information 2). Because of the right-handed chirality, 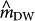 is expected to be oriented outward, as illustrated in the inset. By applying an alternating magnetic field to this bubble domain, unidirectional bubble motion was successfully accomplished, as seen in Figs. 2a–c. The exact conformity of these images with Fig. 1 proves the principle of the present scheme.

### Speed of bubble motion

The speed *V_B_* of the bubble motion follows the average rate of DW motion under the alternating magnetic field pulses. The forward and backward motions of the DW (blue arrows in Figs. 2b and c) yield the relation *V_B_* = [*V*_||_(*H_z_*,*H_x_*) + *V*_||_(−*H_z_*,−*H_x_*)]/2, where *V*_||_ is the DW speed at the rightmost point of the bubble^25^. The measured *V_B_* is plotted with respect to *H_x_* for several *H_z_* (Fig. 2d). This plot clearly demonstrates that *V_B_* is proportional to *H_x_* within the experimental range of *H_x_*, yielding the expression *V_B_* ≅ *ρ*_1_(*H_z_*)*H_x_* (see Supplementary Information 3). According to Ref. 25, the coefficient *ρ*_1_(*H_z_*) is given by {*C*_1_ln[*V*_0_/|*V*_||_(*H_z_*,0)|]}*V*_||_(*H_z_*,0) in the DW creep regime^28^,^29^, where *C*_1_ is a constant related to the Zeeman contribution to the DW energy and *V*_0_ is the characteristic speed. In the present sample, *C*_1_ln[*V*_0_/|*V*_||_(*H_z_*,0)|] is estimated to be approximately (86 mT)^−1^ for the maximum *H_z_* (68 mT) from the present coil and thus, a *V_B_* ( = 1 m/s) is achieved up to approximately 46% of *V*_||_(*H_z_*,0) ( = 2.2 m/s) under the maximum *H_x_* (40 mT).

### Bubble radius variation

**Figure 3:**
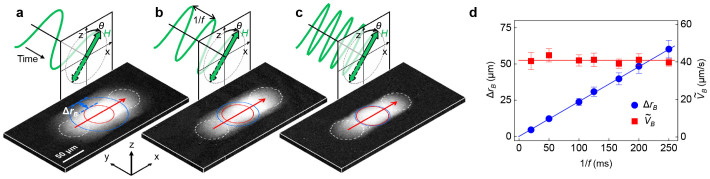
Frequency dependence of bubble motions. Accumulated MOKE images acquired during bubble motion for various *f* values, 10 Hz (a), 20 Hz (b), and 50 Hz (c), respectively. Alternating sinusoidal magnetic field was applied with an amplitude 3.7 mT and a tilting angle 34°, and the accumulation time was fixed to 3 s for all images. The red (blue) circle represents the smallest (largest) bubble observed during the motion. The blue arrows indicate Δ*r_B_* between the largest and smallest bubbles. The red arrows indicate the displacement of the bubble center between the initial and final positions (white dashed circles) over the accumulation time. 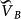 is then determined by the ratio of the displacement over the accumulation time. Since these images were captured over the same accumulation time, almost the same displacements indicate that 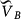 nearly unchanged irrespective of *f*. (d), Measured Δ*r_B_* and 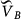 values with respect to *f*. The error bars correspond to the standard deviation from data obtained by sampling 10 times.

The variation Δ*r_B_* in the radius of the bubble during its motion can be controlled by adjusting the frequency *f* of the alternating magnetic field. Figures 3a–c present images of the bubble motion driven by alternating sinusoidal magnetic field with *f* = 10 Hz (a), 20 Hz (b), and 50 Hz (c), respectively. Note that each image was accumulated over a period of 3 s during bubble motion and thus, the length (red arrow) of the gray area represents the bubble displacement during the image-accumulation time. Additionally, the width (blue arrow) of the light-gray boundary represents Δ*r_B_* between the smallest (red circle) and largest (blue circle) bubbles. Figure 3d provides a plot of the measured Δ*r_B_* and the average bubble speed 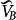 values with respect to *f*. The figure clearly demonstrates that Δ*r_B_* is inversely proportional to *f*. Because 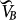 remains nearly unchanged irrespective of *f*, one can independently reduce Δ*r_B_* down to the limiting value defined by the bandwidth of the coil without changing 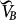.

### Bubblecade and memory operation

**Figure 4:**
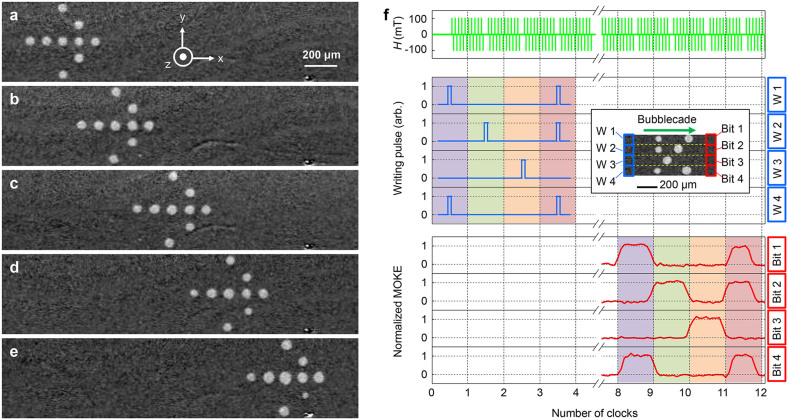
Experimental demonstration of ‘bubblecade', the two-dimensional coherent unidirectional motion of multiple bubbles. MOKE images of an arbitrary 5 × 5 array of bubbles, (a) initially written on the leftmost side of the film and taken after application of (b) 32, (c) 64, (d) 96, and (e) 127 pulse sets. Each pulse set is composed of alternating square magnetic field pulses (*H* *= * ±106 mT, *θ* = 71°, Δ*t* = 20 ms). (f) The operation timetable of 4-bit magnetic bubblecade memory. The top panel presents the alternating magnetic field pulses (*H* = ±106 mT, *θ* = 71°, Δ*t* = 20 ms) that act as the operation clock. The next four panels show the pulses for the thermomagnetic writing of the bubbles on each bit in the writing section. The last four panels show the signals from the CCD pixels corresponding to each bit of the reading section. The inset presents the device structure, with the writing (blue box) and reading (red box) sections indicated.

Finally, the present scheme was applied to a two-dimensional bubble array. For this purpose, an arbitrary 5 × 5 array pattern of bubbles (Fig. 4a) was initially created on the film using the thermomagnetic writing method (see Supplementary Information 4). Under the application of alternating magnetic pulses, all bubbles exhibited coherent unidirectional motion, as shown by the image (Figs. 4b–e and see Supplementary Movie 1) captured during the pulses. Exactly the same bubble-array pattern was maintained even after traveling more than 1 mm (Fig. 4e). Therefore, the observed two-dimensional coherent unidirectional motion of the bubbles—hereafter referred to as the bubblecade—can be used to replace the mechanical motion of the magnetic media with respect to read and write sensors, enabling a new device prototype ‘magnetic bubblecade memory'.

The writing and reading operation schemes of bubblecade memory are also demonstrated. Figure 4f illustrates the operation timetable for a 4-bit magnetic bubblecade memory. The top panel shows the alternating magnetic pulses that act as the operation clock. The next four panels specify the bubble-writing pulses (see Supplementary Information 4), which are applied to each bit of the writing section (blue box) of the device depicted in the inset. At present, bubble writing is achieved using the thermomagnetic writing scheme, but it may also be possible to implement using the spin-transfer torque writing scheme with a nanopillar structure^18^. The bottom four panels illustrate the reading signal from each bit of the reading section (red box) of the device. At present, the reading signals are detected by the corresponding pixels of a charge-coupled device (CCD) camera, but it may also be possible to read out these signals using tunneling magnetoresistive sensors in the future^30^. The figure clearly shows that all written two-dimensional data bits are successively retrieved from the reading section, demonstrating shift-register-based memory operation (see Supplementary Movie 2).

## Discussion

Further optimizing the design of the coils will enhance the operation speed of the magnetic bubblecade memory, because the present maximum of the bubblecade speed is not limited by the sample. Since it is possible to achieve the bubblecade speed to be about a half of the DW speed and the DW speed has been demonstrated to reach a few hundreds m/s (Refs. 4, 6), it is expected to achieve the speed compatible to the practical operations with elaborated design of microcoils. The data rate is then governed by the bubblecade speed and the storage density. Recent discovery of the skyrmion crystals signals the possibility to enhance the storage density of the present scheme up to the packing density of the skyrmions in the skyrmion lattice^19^, which is possibly optimized to be larger than a few hundreds of Gbits/in^2^ with the typical skyrmion size smaller than a few tens of nm (Ref. 18). Furthermore, the two-dimensional bubblecade operation on unpatterned films enhances the compatibility toward the three-dimensional data storage by stacking the films. For better scalability, the stray-field-induced crosstalk and the size-dependence of the bubblecade speed have to be further investigated by exploring the proper materials and layer combination^31^ including the antiferromagnetically coupled layer structures.

In summary, we present here a proof-of-principle experiment demonstrating the two-dimensional coherent unidirectional motion of multiple bubble domains, of which the speed is a significant fraction of the DW speed. Such bubble motion is attributed to the helical magnetic configuration caused by the asymmetric layer structure and therefore, further exploration of materials and layer combinations^31^ together with the optimization of the coil design will further enhance the potential of this technology for various applications. The present scheme can eliminate the necessity for the mechanical motion of the media in hard-disk drives and thus, enables the development of a new prototype solid-state data-storage device, so-called the magnetic bubblecade memory.

## Supplementary Material

Supplementary Information Supplementary Information

Supplementary Information Supplementary Movie 1

Supplementary Information Supplementary Movie 2
