# Supplementary material for: Magnetic bubblecade memory based on chiral domain walls
Source: Sci Rep. 2015 Mar 16;5:9166. doi: 10.1038/srep09166 (PMC5390916; doi:10.1038/srep09166)
Supplement: Supplementary Information [file srep09166-s1.pdf]

Magnetic bubblecade memory based on chiral domain walls

Kyoung-Woong Moon, Duck-Ho Kim, Sang-Cheol Yoo, Soong-Geun Je, Byong Sun Chun, Wondong Kim, Byoung-Chul Min, Chanyong Hwang & Sug-Bong Choe

1. Sample preparation

For this study, metallic ferromagnetic Ta/Pt/Co/Pt films were deposited on Si substrates with 100-nm-thick SiO_2 layer by means of the dc-magnetron sputtering. The thicknesses of the Ta and Co layers are fixed to 5.0 and 0.3 nm, respectively, and the thicknesses of the upper and lower Pt layers are adjusted from 1.0 to 3.0 nm to tune the magnetic properties[31]. To enhance the sharpness of the layer interfaces, the films were deposited with a small deposition rate (0.25 Å /sec) through adjustment of the Ar sputtering pressure (~2 mTorr) and power (~10 W). All the films exhibit clear circular domain expansion with weak pinning strength. The results in Figs. 2 and 3 were obtained from 5.0-nm Ta/2.5-nm Pt/0.3-nm Co/3.0-nm Pt film (Sample A) that shows the fastest bubble speed under the present experimental condition, possibly due to the weak coercive field (7.1 mT). The results in Fig. 4 were obtained from 5.0-nm Ta/2.5-nm Pt/0.3-nm Co/1.0-nm Pt film (Sample B) that allows regular bubble-array writing with small irregularities due to the relatively large coercive field (16.2 mT). The DMI-induced magnetic field H_{DMI} was measured to be 40 and 22 mT for Samples A and B, respectively, by analyzing the asymmetric DW motion[25,26]. The DMI constant is then estimated to be about 0.3 and 0.1 mJ/m^2 for Samples A and B, respectively, by use of the saturation magnetization (1.3×10^6 A/m) measured by a vibrating sample magnetometer and the typical DW width (5 nm).

2. Experimental setup and procedure

The magnetic domain images were observed by use of a magneto-optical Kerr effect (MOKE) microscope equipped with a charge-coupled device (CCD) camera on the focal plane[10]. To

apply the magnetic field onto the films, two electromagnets and two small coils are attached to the sample stage. One of the electromagnets is used to apply the in-plane magnetic field bias up to 200 mT. The smallest coil (~1 mm in radius) is used to apply the out-of-plane magnetic field pulses up to 68 mT with a fast rising time (< 1 μs). The combination of the in-plane electromagnet and the smallest coil was used to obtain the results shown in Fig. 2. The other coil (~2 mm in radius) is designed to apply the alternating sinusoidal magnetic field with adjustable tilting angle, which was used to obtain the results shown in Fig. 3. For field uniformity over the wide range (> 2 mm) of the film, two electromagnets were used to apply the in-plane and out-of-plane magnetic fields to obtain the results shown in Fig. 4.

3. Speed of the bubblecade motion

By adopting the Taylor expansion with respect to H_x, the DW speed $V_{\parallel}$ at the rightmost point of the bubble domain can be written as

$$V_{\parallel}(H_z, H_x) = V_{\parallel}(H_z, 0) + \sum_{n=1}^{\infty} \rho_n(H_z) H_x^n, \tag{S1}$$

where $\rho_n(H_z) \equiv \frac{1}{n!} \frac{\partial^n V_{\parallel}(H_z,H_x)}{\partial H_x^n}\Big|_{H_x=0}$. Since $V_{\parallel}$ is an odd function with respect to H_z, $V_{\parallel}(-H_z, -H_x)$ is equal to $-V_{\parallel}(H_z, -H_x)$ i.e.

$$V_{\parallel}(-H_z, -H_x) =- V_{\parallel}(H_z, 0) - \sum_{n=1}^{\infty} \rho_n(H_z)(-H_x)^n. \tag{S2}$$

From the relation $V_B = [V_{\parallel}(H_z, H_x) + V_{\parallel}(-H_z, -H_x)]/2$, the speed V_B of the bubble motion can be thus expressed as

$$V_B(H_z, H_x) = \sum_{n=0}^{\infty} \rho_{2n+1}(H_z) H_x^{2n+1}, \tag{S3}$$

The experimental observation (Fig. 2d) indicates that it is good enough to approximate Eq. (S3) as

$$V_B(H_z, H_x) \cong \rho_1(H_z) H_x, \tag{S4}$$

within the present experimental range of H_x, by confirming that the higher-order terms are negligible compared to the linear term.

According to Ref. 25, the DW energy density σ_{DW} is given by a function of H_x as

$$\sigma_{\mathrm{DW}}(H_x) = \begin{cases} \sigma_0 - \frac{\pi\lambda M_{\mathrm{S}}}{2H_{\mathrm{D}}}(H_x + H_{\mathrm{DMI}})^2 & \text{for } |H_x + H_{\mathrm{DMI}}| < H_{\mathrm{D}} \\ \sigma_0 + 2K_{\mathrm{D}}\lambda - \pi\lambda M_{\mathrm{S}}|H_x + H_{\mathrm{DMI}}| & \text{otherwise} \end{cases}, \tag{S5}$$

where σ_0 is the DW energy of the Bloch configuration, λ is the DW width, M_{S} is the saturation magnetization, and H_{DMI} is the DMI-induced effective magnetic field. Here, H_{D} ($\equiv 4K_{\mathrm{D}}/\pi M_{\mathrm{S}}$) is the DW anisotropy field that is required to rotate $\hat{m}_{\mathrm{DW}}$ from the Bloch configuration to the Néel configuration, where K_{D} is the DW anisotropy constant. Based on the assumption that the dependence of $V_{\parallel}$ on H_x is solely attributed to the variation of σ_{DW} due to H_x, one finds the relation

$$\rho_1 \equiv \left.\frac{\partial V_{\parallel}}{\partial H_x}\right|_{H_x=0} = \left.\frac{\partial V_{\parallel}}{\partial \sigma_{\mathrm{DW}}}\right|_{\sigma_{\mathrm{DW}}=\sigma_{\mathrm{DW}}(0)} \cdot \left.\frac{d\sigma_{\mathrm{DW}}}{dH_x}\right|_{H_x=0}, \tag{S6}$$

which is then written as

$$\rho_1 = \begin{cases} -\pi\lambda M_\mathrm{S} \frac{H_\mathrm{DMI}}{H_\mathrm{D}} \frac{\partial V_\parallel}{\partial \sigma_\mathrm{DW}}\Big|_{\sigma_\mathrm{DW}=\sigma_\mathrm{DW}(0)} & \text{for } |H_\mathrm{DMI}| < H_\mathrm{D} \\ -\pi\lambda M_\mathrm{S} \mathrm{sgn}(H_\mathrm{DMI}) \frac{\partial V_\parallel}{\partial \sigma_\mathrm{DW}}\Big|_{\sigma_\mathrm{DW}=\sigma_\mathrm{DW}(0)} & \text{otherwise} \end{cases}, \quad \text{(S7)}$$

where $\mathrm{sgn}(H_\mathrm{DMI})$ denotes the sign of H_DMI.

In the creep regime, $V_\parallel$ follows the creep scaling law $V_\parallel(H_z, H_x) = V_0 \exp\left[-\alpha(H_x) H_z^{-1/4}\right]$, where V_0 is a characteristic speed and α is a constant related to the scaling energy constant, the critical magnetic field, and the thermal fluctuation energy[S1]. By use of the relation $\alpha(H_x) \propto [\sigma_{DW}(H_x)]^{1/4}$ proposed in Refs. 25 and 26, one finds the relation

$$\frac{\partial V_\parallel}{\partial \sigma_\mathrm{DW}}\Big|_{\sigma_\mathrm{DW}=\sigma_\mathrm{DW}(0)} = \frac{1}{4\sigma_\mathrm{DW}(0)} V_\parallel(H_z, 0) \ln\left(\frac{V_0}{|V_\parallel(H_z,0)|}\right). \quad \text{(S8)}$$

Since $|H_\mathrm{DMI}| > H_\mathrm{D}$ in the present Pt/Co/Pt films as demonstrated in Ref. 11 and $H_\mathrm{DMI} > 0$ in the present experimental condition, V_B can be finally written as

$$V_B(H_z, H_x) \cong C_1 \ln\left(\frac{V_0}{|V_\parallel(H_z,0)|}\right) V_\parallel(H_z, 0) H_x, \quad \text{(S9)}$$

where $C_1 \equiv \frac{\pi\lambda M_S}{4\sigma_\mathrm{DW}(0)} \mathrm{sgn}(H_\mathrm{DMI})$.

For alternating sinusoidal magnetic field $(H_z \sin\omega t, H_x \sin\omega t)$, the average speed $\tilde{V}_B$ of the bubble motion can be written by

$$\begin{aligned} \tilde{V}_B(H_z, H_x) &\cong \frac{C_1\omega}{\pi} \int_0^{\pi/\omega} \ln\left(\frac{V_0}{|V_\parallel(H_z \sin\omega t, 0)|}\right) V_\parallel(H_z \sin\omega t, 0)\, H_x \sin\omega t\, dt \\ &\cong C_2 V_B(H_z, H_x) \end{aligned}, \quad \text{(S10)}$$

where $C_2 = \frac{1}{\pi}\int_0^{\pi} \exp\left[\ln\left(\frac{V_0}{|V_\parallel(H_z,0)|}\right)\left(1-(\sin\tau)^{\frac{1}{4}}\right)\right](\sin\tau)^{\frac{3}{4}} d\tau$. Numerical evaluation reveals that C_2 is a slowly-varying function of $V_\parallel(H_z,0)$ within the range from 0.3 (for $V_\parallel(H_z,0) \cong$ 1 mm/s) to 0.5 (for $V_\parallel(H_z,0) \cong$ 100 m/s) in the present samples.

According to Eq. (S9), the direction of the bubble motion i.e. $\mathrm{sgn}(V_B)$ is given as

$$\mathrm{sgn}(V_B) = \mathrm{sgn}(H_{\mathrm{DMI}} V_\parallel(H_z,0) H_x). \tag{S11}$$

Since the direction of $V_\parallel$ is determined by the relative alignment of the out-plane magnetic field H_z with respect to the out-of-plane component m_z^{bubble} of the magnetization inside the bubble domain, one can replace $\mathrm{sgn}(V_\parallel(H_z,0))$ by $\mathrm{sgn}(m_z^{\mathrm{bubble}} H_z)$. Then, $\mathrm{sgn}(V_B)$ can be rearranged as

$$\mathrm{sgn}(V_B) = \mathrm{sgn}(\kappa_{\mathrm{DMI}})\cdot\mathrm{sgn}(\theta), \tag{S12}$$

where the DW chirality κ_{DMI} is defined by $H_{\mathrm{DMI}} m_z^{\mathrm{bubble}}$ inside the DW at the rightmost point of the bubble domain and the tilting angle θ of the magnetic field is defined by $\mathrm{atan}(H_x/H_z)$.

We confirmed the dependence on $\mathrm{sgn}(\theta)$ by a repeated experiment with opposite sign of θ (not shown). Such dependence on $\mathrm{sgn}(\theta)$ can be also verified even in the present experimental results, by rotating the observation coordinate by 180 degree with respect to the *z* axis. On the other hand, to confirm the dependence on κ_{DMI}, we repeated the experiment by use of Pt/Co/MgO films that are known to have the left-handed chirality[S2], opposite to the right-handed chirality in the Pt/Co/Pt films[25,26]. Figure S1 summarizes the results from the Pt/Co/MgO films. The results truly show that the direction of the bubble motion in the Pt/Co/MgO films is opposite to that of the Pt/Co/Pt films (Fig. 2), verifying Eq. (S12).

4. Thermomagnetic writing of bubble domains

To create bubble domains, the thermomagnetic writing scheme[S1,S3] is adopted. In this scheme, the magnetization of the film is first saturated by applying an out-of-plane magnetic field pulse (–30 mT, 1 s). A laser beam (60 mW) is then focused on a small spot (~1 μm in diameter) of the film, causing reduction of the coercive field inside the spot by increasing the temperature. At this instant, a reversed magnetic field pulse (8 mT, 12 ms) is applied. Since the strength of the reversed magnetic field is adjusted to be slightly larger than the reduced coercive field inside the spot, the magnetization reversal occurs only in the area of the spot. Consequently, a bubble-shaped reversed domain is created. By repeating this procedure with motorized sample stage, arbitrary pattern of bubble domain array can be recorded. Due to the time delay for multiple-bubble recording with stage translation, the clock pulses were interleaved in the present demonstration of the magnetic bubblecade memory operation shown in Fig. 4f. The spin-transfer torque scheme with multiple nanopillar structures will possibly provide a parallel writing capability required for real-time device operation.

References

S1. Kim, K.-J. *et al*. Interdimensional universality of dynamic interfaces. *Nature* **458,** 740-742 (2009).

S2. Emori, S., Bauer, U., Ahn, S.-M., Martinez, E. & Beach, G. S. D. Current-driven dynamics of chiral ferromagnetic domain walls. *Nat. Mater*. **12,** 611-616 (2013).

S3. Moon, K.-W. *et al*. Distinct universality classes of domain wall roughness in two-dimensional Pt/Co/Pt films. *Phys. Rev. Lett.* **110,** 107203 (2013).

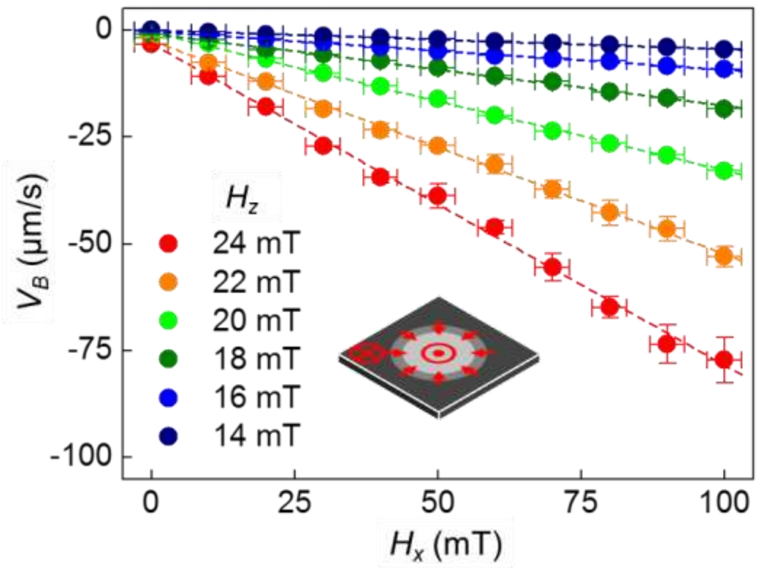

Figure S1 | V_B in Pt/Co/MgO film, with respect to H_x for several H_z. The inset illustrates the expected $\hat{m}_{\mathrm{DW}}$ (red arrows) with the left-handed chiral DW configuration. The dashed lines show the best linear fit from Eq. (S9).

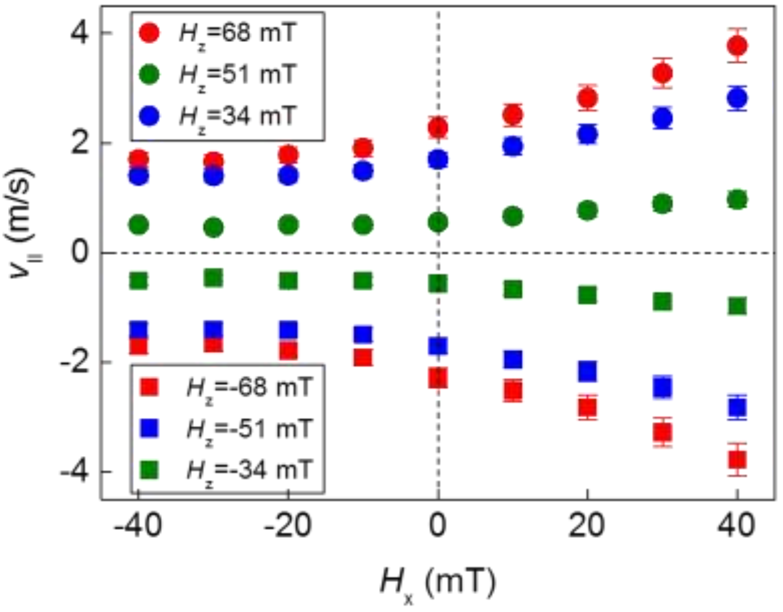

Figure S2 | Asymmetric DW speed $V_{\parallel}$ with respect to H_x for several H_z. Data were obtained under the same condition employed to measure Fig. 2d. The asymmetry with respect to H_x is known to be caused by a finite DMI[25,26]. For the condition (with $H_z = 68$ mT and $H_x = 40$ mT) of the maximum V_B shown in Fig. 2d, the forward and backward DW speeds are measured as $V_{\parallel}(H_z, H_x) = 3.7$ m/s and $V_{\parallel}(-H_z, -H_x) = -V_{\parallel}(H_z, -H_x) = -1.6$ m/s. These speeds are accordant to the measured maximum V_B (=1 m/s) in Fig. 2d via the relation $V_B = [V_{\parallel}(H_z, H_x) + V_{\parallel}(-H_z, -H_x)]/2$. This maximum V_B is approximately 46% of $V_{\parallel}(H_z, 0)$ (=2.2 m/s).
